# The right ventricle: always normal in normal subjects?

**DOI:** 10.1007/s12471-014-0640-y

**Published:** 2014-12-10

**Authors:** E. E. van der Wall

**Affiliations:** Holland Heart House/Netherlands Society of Cardiology, Moreelsepark 1, 3511 EP Utrecht, the Netherlands

Right ventricular (RV) structure and function serve as important indicators of a wide spectrum of heart diseases such as congenital heart disease, pulmonary hypertension, hypertrophic cardiomyopathy and arrhythmogenic right ventricular cardiomyopathy/disease (ARVC/D) [1–3]. Over the past years cardiac magnetic resonance imaging (CMR) has proven itself as the preferred imaging modality of choice to evaluate RV anatomy, structure, texture, motion and function [4–9]. In the study by Quick et al. [10], published in the current issue of the Netherlands Heart Journal, a quantitative analysis of normal variations in RV wall motion was evaluated in healthy subjects using CMR. The study population consisted of 65 consecutive patients referred for the evaluation of cardiac function by 3 Tesla-CMR. All subjects were shown to be free of heart disease based on the currently available guidelines for CMR. Balanced steady-state free-precession images were obtained and regional RV wall motion was evaluated and classified based on a standardised segmental model for the right ventricle. In 59 out of 65 subjects (>90 %) wall motion abnormalities of the right ventricle were observed. Wall motion abnormalities were predominantly seen in the apico-lateral segments (72 %) when compared with the medio-lateral (24 %) and infero-lateral segments (4 %). Dyskinesia was the most frequent wall motion disorder (62 %), followed by hypokinesia (21 %) and bulging (17 %). The authors concluded that RV wall motion abnormalities are common in subjects supposed to be normal, indicating that one should be aware of the notion that non-pathological wall motion disorders can easily be mistaken for a pathological regional wall motion abnormality, particularly in patients with ARVC where –according to the authors- to date, clear wall motion criteria are lacking.

The study is certainly very interesting in the sense that the right ventricle in normal subjects does not always appear normal when visualised by CMR. Especially, regional wall motion abnormalities are predominantly seen in the apico-lateral segments. However, the study warrants the following three comments. First, also acknowledged by the authors, the study was based on a small patient population. As a result, the description of normal variations in RV morphology as observed by CMR are not necessarily absolute and should only be regarded as hypothesis-generating for studying larger patient samples. Second, according to the authors, there is supposedly no in-depth knowledge on RV motion in healthy subjects. This is an obvious understatement as already in 2004 Pennell’s group (London, UK) showed that CMR demonstrated good inter-study reproducibility for RV function parameters in 20 healthy subjects, in 20 patients with heart failure, and in 20 patients with hypertrophy, suggesting that CMR is reliable for serial RV assessment [11]. In 2008, Youssef et al. [12] showed in 21 healthy subjects that the strain-encoding CMR technique allowed for rapid quantification of RV regional function with low intra- and inter-observer variability, permitting accurate quantification of regional strain in patients with RV dysfunction. Rather recently, Von Knobelsdorff-Brenkenhoff et al. [13], in nine healthy subjects, showed that fast-gradient echo cine imaging of the RV at 7 Tesla-CMR was feasible and provided good image quality; RV dimensions and function were comparable with steady-state free-precession at 1.5 Tesla-CMR as gold standard. Doesch et al. [14] showed in 20 healthy subjects that CMR-derived measurement of the tricuspid annular plane systolic excursion with a reference point outside the ventricle (TAPSEout) might be used for screening RV motion; however, the detection of subtle changes in RV function requires the 3D volumetric CMR approach. Third, and most importantly, Quick et al. [10] rather firmly state, that -particularly in ARVC patients- ‘to date clear wall motion criteria are lacking’. However, the revised ARVC/D diagnostic Task Force Criteria, already dating from 2010, incorporate clear CMR-defined cut-off values for RV ejection fraction and RV end-diastolic volume for ARVC [3, 15]. More specifically, the 2010 ARVC/D Task Force Criteria include the following parameters for CMR: 1) regional RV akinesia or dyskinesia or dyssynchronous RV contraction, 2) ratio of RV end-diastolic volume to BSA ≥110 mL/m2 (male) or ≥100 mL/m2 (female), or RV ejection fraction ≤40 %. Used in this way, CMR-derived RV ejection fraction can help in distinguishing ARVC/D from both normal subjects and/or from the physiological cardiac adaptation in athletes [3, 15].

To summarise, despite the above-mentioned critical comments, the study by Quick et al. [10] is a valuable contribution to our understanding of CMR-derived RV function in normal subjects. The study clearly shows that RV wall motion abnormalities may occur both in normal subjects and in diseased patients, in particular those with ARVC. Fortunately, CMR-derived parameters, as defined by the 2010 ARVC/D Task Force Criteria, may provide a major discriminatory role.
